# Structural and Energetic Characterization of the Denatured State from the Perspectives of Peptides, the Coil Library, and Intrinsically Disordered Proteins

**DOI:** 10.3390/molecules26030634

**Published:** 2021-01-26

**Authors:** Elisia A. Paiz, Karen A. Lewis, Steven T. Whitten

**Affiliations:** Department of Chemistry and Biochemistry, Texas State University, San Marcos, TX 78666, USA; elisia.paiz@utsouthwestern.edu (E.A.P.); karen.lewis@txstate.edu (K.A.L.)

**Keywords:** denatured state ensemble, protein coil library, peptides, intrinsically disordered proteins

## Abstract

The α and polyproline II (PPII) basins are the two most populated regions of the Ramachandran map when constructed from the protein coil library, a widely used denatured state model built from the segments of irregular structure found in the Protein Data Bank. This indicates the α and PPII conformations are dominant components of the ensembles of denatured structures that exist in solution for biological proteins, an observation supported in part by structural studies of short, and thus unfolded, peptides. Although intrinsic conformational propensities have been determined experimentally for the common amino acids in short peptides, and estimated from surveys of the protein coil library, the ability of these intrinsic conformational propensities to quantitatively reproduce structural behavior in intrinsically disordered proteins (IDPs), an increasingly important class of proteins in cell function, has thus far proven elusive to establish. Recently, we demonstrated that the sequence dependence of the mean hydrodynamic size of IDPs in water and the impact of heat on the coil dimensions, provide access to both the sequence dependence and thermodynamic energies that are associated with biases for the α and PPII backbone conformations. Here, we compare results from peptide-based studies of intrinsic conformational propensities and surveys of the protein coil library to those of the sequence-based analysis of heat effects on IDP hydrodynamic size, showing that a common structural and thermodynamic description of the protein denatured state is obtained.

## 1. Introduction

Proteins under biological conditions exhibit marginal structural stability [1], and they unfold and refold repeatably in vivo [2]. Consequently, many of the biological processes that are facilitated by protein macromolecules are modulated by the properties and energetic character of the denatured state. Indeed, numerous efforts have shown that denatured state effects, such as residual structure [3], excluded volume [4], and intrinsic conformational propensities [5], have key roles in molecular recognition [6], allosteric signaling [7], folding [8,9], and stability [10]. A molecular-level understanding of how proteins are utilized for biological work thus requires characterization of the native, as well as the myriad of non-native, conformational states that exist in solution for a protein, the latter of which is referred to as its denatured state ensemble (DSE).

Despite its importance in understanding protein function, the probability and structural character of the full spectrum of states sampled by proteins are not known. Numerous studies have used short peptides as experimental models from which to probe the characteristics of the DSE [11,12,13]. The use of short peptides is advantageous because, being too short to fold, they offer access to unfolded states under otherwise folding conditions. Moreover, in the absence of folding, conformational preferences are simplified and locally driven by factors such as hydration [14] and steric hindrance [15]. These studies find that peptides have strong preferences for the polyproline II (PPII) backbone conformation, even at nonproline positions [12,16,17], suggesting that PPII structures are dominant components of the DSE. The PPII conformation is characterized by an extended left-handed helical turn with the amide hydrogen and the carboxyl oxygen of each peptide backbone projecting into solution, presumably making favorable contact with the solvent [18,19,20]. In addition, the PPII conformation appears to facilitate favorable intrachain n→π* interactions, which should be a stabilizing factor [21]. Short peptides also exhibit conformational preferences for other backbone structures. At cold temperatures, alanine residues have intrinsic α-helix-forming tendencies (i.e., even in the absence of favorable side chain interactions) that are stabilized predominantly by peptide hydrogen bonds [22]. Elevated temperatures have been observed to promote low levels of β-strand [16] or β-turn [23], though the amino acid preferences for forming strand [24] or reverse turn structures [25,26] are thought to be highly context-dependent.

The protein coil library [27] also has been used as a structural model for the DSE [28,29,30]. These libraries are constructed from the segments of protein structure in the Protein Data Bank (PDB) that are found outside the α-helix and β-strand domains. Some libraries further omit additional conformationally restricted positions, such as those in reverse turns, or preceding prolines, or immediately flanking a region of secondary structure [29]. The underlying assumption when using a coil library as a DSE model is that site-specific effects on the intrinsic conformational preferences of the amino acids are minimized by averaging over many environments, and also by removing the regular and repetitive interactions associated with folded structures. Overall, coil libraries exhibit structural trends that are in good agreement with the results from peptide structural studies [29,31]. For example, chemical shifts and three-bond *J* couplings (^3^*J_HNα_*) measured in peptides by NMR spectroscopy can be reproduced from structural models made from the protein coil library [32,33,34]. Notably, and similar to the results obtained from peptides, strong preferences for PPII that vary by amino acid type are found in structural surveys of the protein coil library [28,29,30].

Intrinsically disordered proteins (IDPs) offer another experimental system from which to assess structural preferences in unfolded states under nondenaturing conditions [35]. While chemically denatured proteins are known to adopt macromolecular sizes that depend weakly on sequence details other than chain length [36], IDPs in water exhibit strong sequence-dependent influences on structural size [37]. Computer simulations show that steric effects on disordered structure cannot account for the hydrodynamic size dependence on sequence observed in IDPs [38]. Additionally, temperature changes are found to induce large shifts in the hydrodynamic size for disordered proteins in water [39,40,41] that can exceed the change in size associated with the heat denaturation of folded proteins of the same chain length [42]. The implication of these findings, albeit expected, is that monomeric disordered protein structure is both under thermodynamic control and highly sensitive to the primary sequence.

In this review, we show that the sequence dependence of IDP hydrodynamic size can be described from the amino acid-specific biases for PPII in the denatured state. Because PPII-rich structures are extended [43], the magnitude of a PPII preference in the denatured state can affect its mean hydrodynamic size [44,45]. Specifically, experiments that evaluate how IDP hydrodynamic size changes with compositional changes in the protein give an independent measure of PPII bias, and further reveal amino acid-specific preferences for PPII that are in good quantitative agreement with PPII bias determined experimentally in peptides [37]. Good agreement is also found when the IDP results are compared to PPII bias in the protein coil library. Moreover, the analysis of heat effects on IDP hydrodynamic size indicates the PPII bias is driven by a significant and favorable enthalpy, and is partially offset by an unfavorable entropy [37], which, again, agrees quantitatively with the peptide results [46]. Across these three models (i.e., peptides, the coil library, and IDPs), the data indicate that the structural and energetic character of the DSE at normal temperatures follows the predictions of a PPII-dominant ensemble. At cold temperatures, both peptides and IDPs reveal the DSE can shift in population toward the α-helix backbone conformation. To demonstrate these conclusions, the following sections review results obtained from numerous spectroscopic and calorimetric studies on short peptides [11,12,13,16,17,46], surveys of structures in the protein coil library [28,29,30], and the more recently acquired sequence- and temperature-based analysis of IDP hydrodynamic sizes [37], showing that these three experimental systems used for characterizing unfolded proteins under folding conditions convey a surprisingly consistent structural and energetic view of the DSE.

## 2. Peptide Models of the DSE

**Figure 1:**
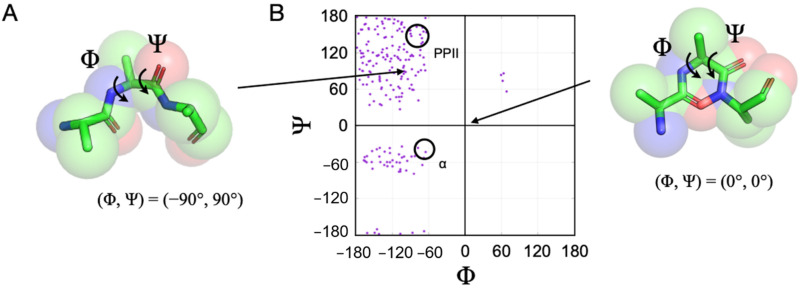
Sterically allowed backbone conformations in polypeptides. (**A**) Peptide backbone dihedral angles, Φ and Ψ. (**B**) Ramachandran plot of allowed Φ, Ψ for the central residue in a poly-alanine tripeptide, calculated from structures generated computationally using a hard sphere collision (HSC) model [48,49] and the “normal” atom pair distances from Ramachandran et al. [47]. Approximately 9000 random structures were generated to find 200 sterically allowed configurations. Highlighted by the circled areas are Φ, Ψ regions corresponding to the PPII and α-helix backbone conformations, as indicated.

The structural preferences associated with unfolded proteins are often described in terms of a predisposition for specific pairs of backbone dihedral angles, phi (Φ) and psi (Ψ). Visually, this is demonstrated with a Ramachandran plot, shown in Figure 1, where pairs of Φ, Ψ angles that are sterically accessible to a polypeptide chain are mapped [47]. For example, using a representative plot computed for the central residue in a poly-alanine tripeptide, it shows that (Φ, Ψ) = (0°, 0°) is found in a disallowed region of the plot because these angles for the central residue place the backbone carbonyl oxygen and backbone nitrogen from the first and third residues, respectively, inside the normal contact limits, creating a steric conflict. In contrast, (Φ, Ψ) = (−90°, 90°) for the central alanine has no such contact violations for any of the tripeptide atoms, and thus this angle pair is physically allowed. When an unfolded protein shows preferences for some allowed Φ, Ψ pairs at the expense of others, specifically during the rapid interconversion between states of its conformational ensemble, it is said that the unfolded protein exhibits a conformational bias.

The idea that unfolded proteins and polypeptides in water may exhibit intrinsic biases for some backbone conformations at the expense of others began to receive widespread consideration when the observation was made that, for a protein chain to achieve its unique structure in a biologically relevant time frame, a random search of all accessible conformations is not possible [50]. The unfolded chain, accordingly, must search a smaller conformational space to what would be predicted from steric considerations alone. This observation predicted that folding is guided by the structural characteristics of the DSE, and experiments to identify folding intermediates, both kinetic [51,52] and equilibrium [53,54], and measure the intrinsic conformational propensities of the amino acids [5] have been extensively pursued over the many decades since.

Early experimental evidence indicating structural preferences in the DSE was provided by Tiffany and Krimm from studies on short poly-proline and poly-lysine peptides using circular dichroism (CD) and optical rotatory dispersion (ORD) spectroscopies [55,56,57]. Though these short peptides were unfolded, owing to insufficient chain length for forming compact, globular structures, Tiffany and Krimm found strong preferences for PPII structures. This structural motif at the residue level corresponds to the *trans* isomer of the peptide bond and (Φ, Ψ) of approximately (−75°, +145°) [43,55]. Its presence in a polypeptide can be established from positive and negative bands in the spectroscopic readings at ~220 nm and ~200 nm, respectively [55,56]. The predisposition for adopting PPII was linked to a variety of factors, such as low temperatures, steric hindrance between side chains, a lack of internal hydrogen bonding, and protonation [57]. Short peptides of poly-glutamic acid also were observed to transition from α-helix at low pH to PPII at neutral pH and higher, identified from CD and ORD spectroscopies [56], indicating that structural transitions between one region of the Ramachandran plot to others could occur for some sequences owing to simple changes in the peptide charge state. These results, Tiffany and Krimm hypothesized, predict a DSE dominated by backbone interconversions between three main structural states: PPII, α-helix, and unordered, where unordered is represented by the random chain [57]. They also speculated, to some resistance [58,59,60], that solvation effects may contribute to the observed PPII preferences, since the PPII configuration places the backbone amide and backbone carbonyl oxygen polar groups in favorable positions for contact with water. Intrinsic PPII propensities thus could be helpful for keeping unfolded proteins solvated. Overall, their findings from these peptide-based studies supported the idea that unfolded proteins, though highly dynamic and exhibiting broad structural heterogeneity, nonetheless can show backbone conformational biases that are determined locally by sequence details.

Peptide studies have also made extensive use of poly-alanine, because of the natural abundance of alanine in proteins and its chemically simple side chain (i.e., a methyl group). Using a peptide called XAO, where A is an alanine heptamer and X and O are flanking diaminobutyric acid and ornithine, respectively, Kallenbach and coworkers found strong, temperature-dependent preferences for the PPII conformation [11]. ^3^*J_HNα_* coupling constants measured by NMR techniques were used to estimate the Φ angle at each alanine position from the Karplus relationship [61], and it was found that Φ was approximately −70° at low temperatures. Because both PPII and α-helix can have Φ angles near this value (Figure 1), the presence of the α-helix was ruled out by a lack of measurable NOEs between successive amides in the peptide chain, which is an indicator for α-helix formation. The CD spectrum of XAO also confirmed PPII content. Increasing temperatures caused gradual reductions in populating the PPII state that coincided with an increasing population of β-strand conformations to approximately 10% at 55 °C. The reduction in PPII content at high temperatures implied a favorable enthalpy of PPII formation that was also observed by Tiffany and Krimm [57]. Further studies of XAO by Asher et al. using UV Raman spectroscopy established that XAO is structurally similar to a 21-residue alanine-peptide, AP, that forms α-helix under cold conditions [62]. AP transitions to PPII at higher temperatures, and demonstrates that AP, similarly to XAO, shows temperature-dependent conformational preferences.

Additional studies that examined a single alanine flanked on both sides by two glycines (i.e., Ac-(Gly)_2_-Ala-(Gly)_2_-NH_2_) found intrinsic preferences for PPII and heat-induced shifts toward β-strand backbone conformations [63]. Temperature-dependent transitions that exhibit similar structural characteristics have also been seen in alanine tripeptides, tetrapeptides, and octapeptides [18,64,65].

To explore the determinants of the PPII bias in greater detail, quantitative studies designed to measure its dependence on amino acid type were initially conducted by Creamer and coworkers [12]. Host–guest substitutions at an internal position in a proline-rich peptide (Ac-(Pro)_3_-X-(Pro)_3_-Gly-Tyr-NH_2_, where X is the substitution site) were used to analyze substitution-induced effects on the CD spectrum and measure a scale of relative PPII propensities for 18 of the 20 common amino acids. Bias estimates for tryptophan and tyrosine were not measured, because the aromatic contribution to the CD spectrum from their side chains overlaps with the region where signal height was used to determine PPII content [66,67], impeding their analysis. These experiments found that amino acids with charged side chains, except for histidine, had relatively high preferences for the PPII conformation in this peptide. The observed biases, measured at 5 °C, were mostly insensitive to changes in solution pH from 2 to 12. Residues with small, non-polar side chains, such as alanine and glycine, reported somewhat higher propensities for PPII that, in general, exceeded the biases observed from residues with non-polar and bulky side chains, such as isoleucine and valine. The list of amino acid-specific intrinsic propensities for PPII determined in these studies is given in Table 1.

Similarly, Kallenbach and coworkers extended their NMR- and CD-based structural studies of the short peptides mentioned above to include other amino acid types at the central residue position in Ac-(Gly)_2_-X-(Gly)_2_-NH_2_, where X was the substitution site. Substitution-induced effects on peptide structure were then used to establish a scale of PPII bias in this glycine-rich host [16]. Substantial intrinsic PPII propensities were found, giving additional support to the idea that unfolded states are predisposed to PPII (see Table 1). The magnitude of the PPII bias at the peptide guest position, surrounded by glycine, however, was noticeably different (and typically larger) when compared to the amino acid-specific biases that were measured in the proline-based host by Creamer. This predicts position-specific PPII bias in an unfolded chain that is modulated by the amino acid identity at neighboring sites, which has been subsequently verified [68]. Moreover, the glycine-rich peptides exhibited a heat-induced shift in structure from PPII to nonPPII with a slight bias at high temperatures for strand-like conformations. The intrinsic PPII propensities reported in Table 1 from Kallenbach were measured at 20 °C. 

A third experimental scale of PPII propensity in peptides was measured calorimetrically by Hilser and coworkers [13,17,69]. Their experiments utilized a peptide host–guest system in which the *Caenorhabditis elegans* Sem-5 SH3 domain binds a peptide in the PPII conformation [70]. This peptide (Ac-Val-(Pro)_3_-Val-(Pro)_2_-(Arg)_3_-Tyr-NH_2_) is derived from the recognition sequence of a SH3 binding partner, Sos (Son of Sevenless). A non-interacting residue of this peptide corresponding to its fourth position [13] was substituted for each amino acid before binding was measured by isothermal titration calorimetry. The observed change in binding affinity reflects a change in the conformational equilibrium between binding-incompetent and binding-competent (i.e., PPII) states of the peptide ligand, which can be interpreted as a PPII propensity [13,69]. Once again, a substantial intrinsic bias for PPII was observed, albeit at magnitudes and rank orders that were different when compared to the scales determined by either Creamer or Kallenbach. Elam et al. conclude that there is a general consensus regarding amino acids that are high in PPII propensity (proline, lysine, glutamine, and glutamic acid) and low in PPII propensity (histidine, tryptophan, tyrosine, and phenylalanine), with the other amino acids falling in between [17]. The intrinsic PPII propensities in Table 1 from Hilser’s group were measured at 25 °C.

There are a number of other studies beyond the few described above, each of which uses their own system to examine the structural propensities of the different amino acids in peptides (reviewed in ref. [71]). While the ranks of relative PPII propensities are often both quantitatively and qualitatively different when compared between studies, possibly owing to the use of different host models, all studies have indicated the same general conclusions that (1) unfolded peptides have structural preferences that are predominantly locally determined [72]; (2) nevertheless, these preferences at individual positions can be modulated by the structural features of neighboring residues [68], and (3) importantly, the unfolded chain does not evenly sample the sterically allowed regions of Ramachandran space [71].

In addition to PPII propensities, alanine-based peptides have been utilized to measure intrinsic α-helix-forming tendencies in a host–guest model that was designed to avoid stabilizing side chain–side chain and side chain–macrodipole interactions [22]. Though cold temperatures were required for this peptide to populate helix at appropriate levels for study, Baldwin and coworkers measured amino acid substitution effects on the CD signal at 222 nm and determined an experimental scale of α-helix intrinsic propensities for each of the 20 common amino acids. At 0 °C, most of the amino acids disfavored forming helix at guest positions in the alanine-based host, while leucine and arginine were indifferent to helix-formation. Alanine, however, had a preference for forming helix in this host. The intrinsic propensity for forming α-helix determined by Baldwin and coworkers for each of the common amino acids is provided in Table 2.

## 3. Protein Coil Library Model of the DSE

The PDB [73] provides an ever-increasing number of high-resolution protein structures, which include both regularly ordered secondary structures (helices, sheets, and turns) and irregularly ordered structures (coils and loops). While any individual coil or loop was sufficiently ordered for structural determination, the assumption is that in aggregate, a large set of irregularly ordered structures would provide information on the conformational tendencies and properties of the polypeptide chain in the denatured state. Collectively, these models of the denatured state are constructed by examining the regions of resolved protein structures that are outside the α-helix and β-strand domains. Indeed, analyses of “protein coil libraries” generally support the structural preferences that have been observed in peptide-based models. As these libraries of coil structures have evolved, the field has gained valuable insights into the roles of sequence context, intramolecular interactions, and protein hydration in determining the intrinsic structural tendencies of the amino acids.

In 1995, Swindells and Thornton generated one of the first iterations of a protein coil library based on high-resolution protein structures [27]. Four basins were defined on the Ramachandran plot, corresponding to a (α-helix), b (β-sheet), p (PPII), and L (left-handed helix). Using 85 structures obtained from the PDB, they removed residues that were assigned helix or sheet conformation, retaining all coils, loops, and turns in the analyzed set. Within this set, residues Glu, Gln, Ser, Asp, and Thr demonstrated strong propensities for the “a” region, as their side chains have both the hydrogen bonding capacity and rotational flexibility to form hydrogen bonds to backbone groups. The “b” propensities appeared to be less sensitive to the chemistry and rotamer of the side chain, consistent with the location of the side chain relative to the backbone when in the β-sheet conformation. While the authors did not explicitly discuss the “p” region (PPII), their data show a significant redistribution of the population between the four basins when the “whole” and “coil library” sets are compared. When the entire polypeptide chain was considered, the a and b basins were the two most highly populated. In the coil library, with helices and sheets removed, the a and p basins exhibited the highest populations. This demonstrated that in the structures of intact proteins, PPII conformations are well represented in the non-alpha and non-beta regions.

This work was followed by an analysis of the PPII content in 274 high-resolution structures conducted by Stapley and Creamer [74]. In their analysis, they found the PPII conformation was common, with more than half of the proteins containing at least one PPII helix longer than three residues, despite PPII residues comprising just 2% of all residues in the dataset. This study was the first to detail the PPII propensities of each side chain. Predictably, Gly was disfavored, while Pro had a strong PPII propensity. Additionally, they observed that Gln, Arg, Lys, and Thr had generally strong propensities for adopting PPII conformations. Moreover, a positional dependence of PPII propensity within the PPII helix was also found. The ability of polar side chains, such as Gln, Lys, and Arg, to form hydrogen bonds with the backbone between *i* and *i* + 1 positions stabilizes the PPII helix. This is consistent with the overrepresentation of Gln, Arg, Lys, and Thr in the first PPII helix position. These data also supported the idea that PPII helices have extensive solvent exposure, as there was a significant negative correlation between nonpolar solvent accessibility and PPII propensity. Taken together, their work demonstrated that both solvent accessibility and the ability to form hydrogen bonds with the backbone were important elements of PPII propensity, consistent with prior work in peptides.

In 2005, Rose and coworkers developed a protein coil library (PCL) that is web-accessible [28]. The PCL becomes updated as the PDB is also updated. This repository of structure elements uses the regular expressions for α-helices and β-sheet and then extracts all non-helix and non-sheet residues from deposited structures that share <90% identity. Note that, as a result, the PCL contains both turns and homologous sequences. Additionally, for structure classification purposes, the PCL divides the Ramachandran plot into 30° × 30° bins, whereby each bin refers to one of 144 different “mesostates”.

An analysis in 2008 by Perskie et al. identified seven naturally clustering basins in a Ramachandran plot of PCL structures [30]. These basins represent the familiar α, β, PPII, αL, and τ (type II’ β-turn) structural motifs, and also a γ basin, for inverse γ turns, and a δ basin that captures residues preceding a proline in proline-terminated helices. This allowed amino acid preferences for the different basins (see Table 2 in ref. [30]) to be determined and studied. For example, solvent–backbone hydrogen bonding, which can favor PPII [14], and side chain–side chain sterics, which for branched amino acids adjacent to proline can favor δ at the expense of β, were found to be crucial determinants of the basin preferences.

To better understand how the conformational preferences of a residue in the denatured state depend on the identity and state of its adjacent (nearest) neighbor, Freed and coworkers constructed an increasingly stringent set of coil libraries [29]. Using 2020 nonhomologous polypeptide chains, the “full” set was defined as the entire polypeptide chain, sans the terminal residues. The first cull of the full set (C_αβ_) removed the α-helix and β-sheet identified residues, similar to the original coil libraries and the PCL described above. This had the effect of reducing the number of residues to 40% of the original. The next restriction additionally removed hydrogen-bonded turns from the set (C_αβt_), slimming the library to 28% of the original. Finally, to produce the most restricted coil library, the authors retained only those residue positions located within contiguous stretches four residues or longer, and which were “internal” to coils. This had the effect of reducing “end bias” from structured regions, which is known to favor PPII at the expense of α and β.

The sequential removal of ordered residues had the overall effect of increasing PPII content and decreasing α populations in the coil library. Specifically, when all structured positions were included, α-helical conformations were the predominant state. Upon removing the α-helix and β-sheet residues—as Swindells and Thornton did a decade prior—the PPII conformation emerged as a major subpopulation. With turns also removed (C_αβt_), the most populated conformation was PPII, and there was a significant reduction in the α population. The dominance of the PPII conformation is not restricted to a particular subset of amino acids, as all 20 amino acids show a considerable propensity to adopt the PPII configuration (Table 3). The most restricted set (with only residues that are well within coil regions) showed little change in the population distribution, with the PPII population continuing to be dominant.

Using the most restricted set, the authors also found that the size of the PPII subpopulation is constant regardless of solvent accessibility [29]. Moreover, PPII is the dominant conformation in all but the 10% most surface-exposed residues. The α-helix dominates in the surface residues, due to the propensity of the polypeptide backbone at the surface to preferentially turn back toward the folded core of the protein. The independence of PPII content and solvent accessibility initially appears to contrast with earlier work with both peptides and earlier versions of PCLs. However, these results can be reconciled by understanding the PPII conformation as a mechanism for maximizing backbone hydrogen bonding. In the PPII conformation, the backbone amides and carbonyls are in positions that both minimize steric hindrance and enable both functional groups to form hydrogen bonds, either with solvent molecules or within the protein [29]. Therefore, the PPII propensity likely reflects the intrinsic hydrogen bond capacity of a polypeptide, not merely solvation.

**Figure 2:**
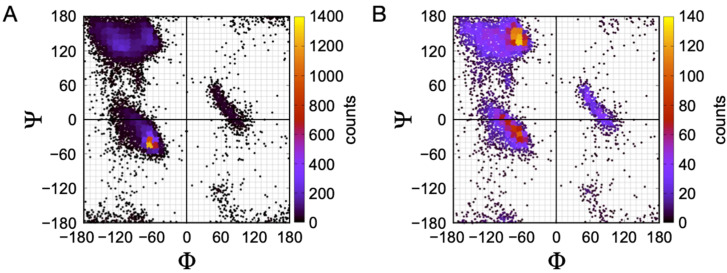
Protein coil libraries are dominated by PPII conformations. In total, 122 non-homologous human structures were analyzed for individual residue conformations (including Gly). (**A**) Ramachandran plot for every residue in the set (15,598 residues). The major population is in the α region, centered at (−65°, −45°). (**B**) Ramachandran plot of the same set after removing all identified α-helix and β-sheet residues (identified using the information provided in the PDB structure file header), yielding 6418 remaining. The major population has shifted to the PPII region, and peaks at (−65°, 135°). For both plots, color represents the count in 10° × 10° bins.

These general results can be replicated using almost any set of nonhomologous protein structures. Figure 2 shows results from a curated set of 122 human protein structures, sharing less than 50% sequence identity and with structural resolution < 2.0 Å [75]. In the full set, containing 15,958 residue positions, the α conformation is the most populated (Figure 2A). When α-helices and β-strands are removed, PPII is the most favored conformation for the remaining 6418 residue positions (Figure 2B).

The consistency of PPII propensity in protein coil libraries, especially when viewed in light of hydrogen bonding capacity, therefore predicts that a bias toward PPII conformations is an inherent characteristic of the polypeptide backbone.

## 4. IDP Model of the DSE

The results of many studies (reviewed above) revealed a significant bias toward PPII in the unstructured states of proteins, even when no prolines are present in the sequence. This indicates that the PPII conformation is a dominant component of the DSE, and potentially an important structural descriptor for understanding the properties associated with IDPs and intrinsically disordered regions (IDRs). Although intrinsic PPII propensities have been determined for the common amino acids (see Table 1 and Table 3), the ability of these experimentally determined propensities to quantitatively reproduce ID structural behavior in biological proteins has been difficult to establish.

An experimental system was designed to address this issue and provide an independent measure of the amino acid-specific bias for PPII in IDPs. Based on the hypothesis that the magnitude of a PPII preference in the disordered conformational ensemble can affect its population-weighted hydrodynamic size [41,44,45], it has been shown that intrinsic PPII propensities can be obtained by analyzing the sequence dependence of the mean hydrodynamic radius, *R_h_*, of IDPs [37]. This method relies on two assumptions we demonstrate are reasonable. First, that PPII effects on mean *R_h_* follow a simple power law scaling relationship [41,44,45], and second, that the protein net charge also can influence the hydrodynamic size [38,76]. 

To establish the relationship linking mean *R_h_* to chain bias for PPII in an ensemble, a computer algorithm based on the hard sphere collision (HSC) model was used to generate polypeptide structures through a random search of conformational space [48,49]. The HSC model has no intrinsic bias for PPII, which was demonstrated previously [49], and thus a PPII sampling bias could be added to the algorithm as a user-defined parameter [41].

Briefly, in this model, individual conformers are generated by using the standard bond angles and bond lengths [77], and a random sampling of the backbone dihedral angles Φ, Ψ, and Ω. (Φ, Ψ) is restricted to the allowed Ramachandran regions [78]; the peptide bond dihedral angle, Ω, is given 100% the *trans* form for nonproline amino acids, while prolines sample the *cis* form at a rate of 6–10%, depending on the identity of the preceding amino acid [79]. The positions of side chain atoms are determined from sampling rotamer libraries [80]. Van der Waals atomic radii [47,81] are used as the only scoring function to eliminate grossly improbable conformations. To calculate state distributions typical of protein ensembles, a structure-based energy function parameterized to solvent-accessible surface areas is used to determine the population weight of the generated structures [82,83,84,85,86,87,88,89,90]. Random structures are generated until the population-weighted structural size, <*L*>, becomes stable [41]. *L* is the maximum C_α_–C_α_ distance in a state, and <*L*> is considered stable when its value changes by less than 1% upon a 10-fold increase in the number of ensemble states. <*L*>/2 is used to approximate the mean *R_h_* of an ensemble. 

**Figure 3:**
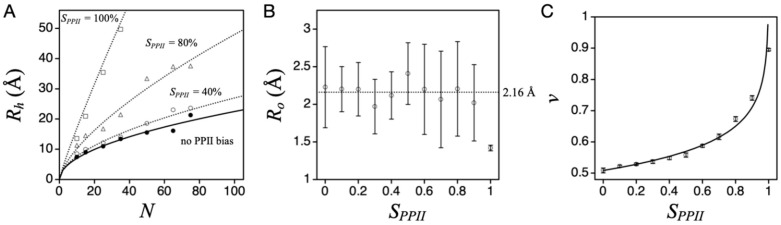
PPII bias expands the structural dimensions of the DSE. (**A**) The effect of an applied PPII sampling bias (*S_PPII_*) on mean *R_h_* (i.e., <*L*>/2) for poly-alanine at different *N*. Filled circles represent no preferential bias, while open circles, triangles, and squares show when *S_PPII_* is 40%, 80%, and 100%, respectively. (**B**) *S_PPII_* effects on fit parameter *R_o_*. Average *R_o_* for *S_PPII_* range of 0–90% is 2.16 Å, indicated by the stippled line. (**C**) *S_PPII_* effects on fit parameter *v* when *R_o_* is held constant at 2.16 Å. Line is from nonlinear least squares fit of these data to the logarithmic equation, *v* = *v_o_* + *a*∙*ln*(1 − *S_PPII_*).

Figure 3A shows the effect on simulated mean *R_h_* (i.e., <*L*>/2) from increasing the applied PPII sampling bias, *S_PPII_*, which is obtained by weighting the random selection of Φ and Ψ. For example, a 30% sampling bias for PPII had 30% of the paired (Φ, Ψ) values for any residue randomly distributed in the region of (−75° ± 10°, +145° ± 10°). The remaining 70% of paired (Φ, Ψ) were distributed in the allowed Ramachandran regions outside of (−75° ± 10°, +145° ± 10°). In this figure, each data point represents a computer-generated poly-alanine conformational ensemble (typically >10^8^ states). These results are mostly insensitive to steric effects originating from the side chain atoms when biological sequences are used instead of poly-alanine [38]. Unusual sequences, such as all proline or all glycine, cause deviations from the poly-alanine trend.

The simulations revealed that increasing chain propensity for PPII gives rise to increased mean *R_h_*, which is expected because PPII is an extended structure [43]. The dependence of mean *R_h_* on chain length at each sampling bias was fit to the power law scaling relationship, *R_h_* = *R_o_*∙*N^v^*, where *N* is chain length in number of residues, *R_o_* the pre-factor, and *v* the polymer scaling exponent. Individual fits at a given *S_PPII_* are shown by lines in Figure 3A, obtained by nonlinear least squares methods. *R_o_*, on average, was 2.16 Å, except when the sampling bias was 100% PPII (Figure 3B). When *R_o_* is held at 2.16 Å, the resulting *v* shows a logarithmic dependence on *S_PPII_* (Figure 3C).

Because most computer-generated random structures have steric conflicts, and thus are removed by the hard sphere filter, the applied PPII bias, *S_PPII_*, does not necessarily equal the population-weighted fractional number of residues in the PPII conformation in an ensemble of allowed states. By using *f_PPII_* = <*N_PPII_*>/*N* to account for this difference, where *N_PPII_* is the number of residues in the PPII conformation in a state, and <*N_PPII_*> is the population-weighted value for the ensemble (i.e., <*N_PPII_*> = ∑*N_PPII,i_*∙*P_i_* with *P_i_* the Boltzmann probability of state *i*), the simulation trends in Figure 3 can be combined into a simple relationship,

(1) $$R_{h}=\left(2.16\;Å\right)\cdot N^{0.503-0.11\cdot ln\left(1-f_{PPII}\right)}$$

Additional simulations found that Equation (1) is independent of the specific pattern of PPII propensities in the polypeptide chain [45].

**Figure 4:**
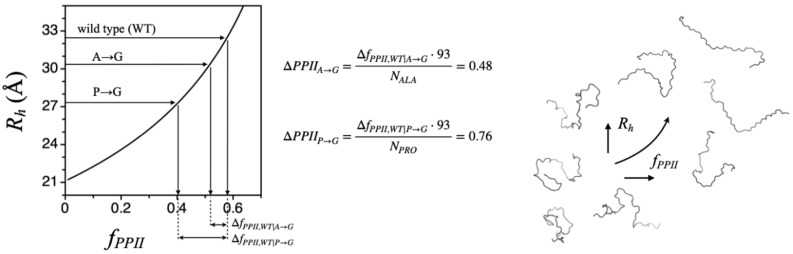
Using mutational effects on IDP *R_h_* to estimate changes in chain bias for PPII. Computed *R_h_* dependence on *f_PPII_* for a 93-residue polypeptide, using Equation (1). Arrows show results from experimental *R_h_* measured by both dynamic light scattering (DLS) and size exclusion chromatography (SEC) methods for wild type p53(1-93) and the P→G and A→G substitution mutants. In total, 22 proline (*N_PRO_*) and 12 alanine residues (*N_ALA_*) in the wild type sequence were substituted to glycine in the P→G and A→G mutants, respectively.

To test Equation (1) directly, mutational effects on experimental *R_h_* were measured for an IDP [44]. Apparent changes in *f_PPII_* were determined from amino acid substitutions, following the strategy shown in Figure 4. These experiments used the N-terminal end of the p53 tumor suppressor protein, a prototypical IDP consisting of 93 residues, p53(1-93). The apparent net charge, *Q_net_*, calculated from sequence for p53(1-93), is −17. Thus, this test was conducted in the background of potentially strong intramolecular charge–charge interactions that were unaccounted for. Nonetheless, experiments with P→G and A→G substitutions applied to p53(1-93) gave reasonable results, indicating a per-position average PPII bias change of 0.76 at each proline site (i.e., relative to the intrinsic PPII bias of glycine) and 0.48 at each alanine site. These results are evidence of significant conformational bias for PPII in IDPs, even at nonproline positions.

**Figure 5:**
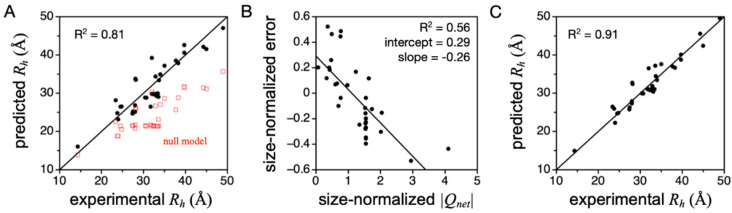
Predicting IDP *R_h_* from sequence using experimental PPII propensities. (**A**) *R_h_* predicted by Equation (1) compared to experimental *R_h_* for 34 IDPs. Predicted values (black circles) were determined from sequence using experimental PPII propensities measured in peptides by Hilser and coworkers (column 4, Table 1). Red squares show *R_h_* predictions when using a null model where PPII is not preferentially populated [45]. (**B**) Size-normalized error, (predicted—experimental *R_h_*)/*N*^0.5^, compared to size-normalized *Q_net_* (i.e., |*Q_net_*|/*N*^0.5^) for each IDP in panel A. (**C**) Equation (2) predicted *R_h_* compared to experimental *R_h_* for 34 IDPs. The identity, primary sequence, and experimental *R_h_* for the IDPs used to generate data in this Figure are provided in ref. [37]. In each plot, R^2^ is the coefficient of determination.

Equation (1) was also used to predict *R_h_* from sequence for a database of IDPs, using the experimental PPII propensities in Table 1 [45]. For each IDP, *f_PPII_* was calculated by ∑ *PPII_i_*/*N*, where *PPII_i_* is the PPII propensity of amino acid type *i*, and the summation is over the protein sequence containing *N* number of amino acids. Figure 5A shows *R_h_* predicted when using PPII propensities from Hilser and coworkers (column 4, Table 1). Compared to the null model where PPII is not strongly preferred and the chain is an unbiased statistical coil, Equation (1) indeed captures the overall experimental trend. Repeating these predictions using the PPII scales measured by Creamer or Kallenbach (columns 2 and 3, Table 1), both yield *R_h_* values that are consistently larger than in the experiment [45], indicating these two scales may be overestimated, at least for describing structural preferences in prototypical IDPs. Moreover, the error from predicting *R_h_* by Equation (1) when using the Hilser-measured PPII scale was found to trend strongly with *Q_net_* when *Q_net_* was normalized to chain length (Figure 5B), more so than >500 other physicochemical properties that can be calculated from the primary sequence [38]. The linear trend in prediction error to *Q_net_* (determined from sequence as number of K and R minus number of D and E) was used to modify Equation (1), yielding

(2) $$R_{h}=\left(2.16\;Å\right)\cdot N^{0.503-0.11\cdot ln\left(1-f_{PPII}\right)}+0.26\cdot \left|Q_{net}\right|-0.29\cdot N^{0.5}$$

Equation (2), which amends Equation (1) for *Q_net_* effects on the hydrodynamic size, is highly accurate in predicting *R_h_* from sequence for many IDPs (Figure 5C). Further, in this set of IDPs, mean *R_h_* did not trend with κ [38], which is a measure of the mixing of positive and negative charges in the primary sequence [91]. This justified using *Q_net_* to modify Equation (1) and obtain Equation (2), because mean *R_h_* was independent of sequence organization of the charged side chains.

**Figure 6:**
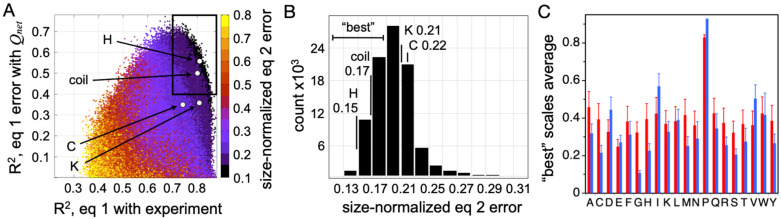
Using experimental *R_h_* from IDPs to determine amino acid-specific intrinsic PPII propensities. (**A**) Ability of experimental PPII propensity scales (from Table 1) to describe the sequence dependence on IDP *R_h_* compared to 10^6^ random PPII propensity scales. Missing amino acids from scales measured by Kallenbach (column 3, Table 1) and Creamer (column 2, Table 1) were given the scale average (bottom value, Table 1). Compared as well is the result from using a coil library scale (Table 3). In panels A and B, results from using scales from Hilser and coworkers, Kallenbach and coworkers, Creamer and coworkers, and the coil library are labeled “H”, “K”, “C”, and “coil”, respectively. (**B**) Histogram of error distribution in the boxed region of panel A. Small errors are better. (**C**) Average scale value calculated for each of the 20 common amino acids using the “best” performing random PPII propensity scales (red bars). Average scale value using the “best” performing random scales that also maintain correct rank order for the nonpolar amino acids (blue bars), yielding an experimental PPII propensity scale based on IDPs. Error bars report standard deviations.

To further test Equation (2) and its ability to describe PPII effects on IDP *R_h_*, random PPII scales were generated and tested for accuracy at predicting experimental *R_h_* [37]—thus establishing the sensitivity of Equation (2) to scale variations. Briefly, each random scale, where the 20 common amino acids were individually assigned random values between 0 and 1, was used to predict *R_h_* by Equation (1), and was then compared to experimental *R_h_*, an example of which is shown Figure 5A for the peptide-based PPII scale measured by Hilser and coworkers. Next, the linear trend in prediction error to size-normalized *Q_net_* was determined, as in Figure 5B. These two steps generate two correlations (R^2^), which were used to evaluate each random scale (Figure 6A). Because the slope and intercept from the error trend with size-normalized *Q_net_* provides the coefficients preceding |*Q_net_*| and *N*^0.5^ in Equation (2), each scale yields a unique empirical modification to Equation (1) that corrects for net charge effects on mean *R_h_*. The results from analyzing 10^6^ randomly generated scales in this manner are given in Figure 6A. Each data point represents a PPII scale. The color, from black to purple, red, and through yellow, is the average error in predicting *R_h_* from sequence after correcting for net charge effects on hydrodynamic size (i.e., after using scale-specific Equation (2) to predict *R_h_*). The abscissa is the correlation (R^2^) of Equation (1)-predicted *R_h_* with the experiment for a scale. The ordinate is the correlation (R^2^) of size-normalized Equation (1) error with size-normalized *Q_net_*.

Two key observations are immediately apparent in the data given in Figure 6A. First, there is a set of random PPII propensity scales that are better than typical at predicting mean *R_h_* from sequence when using *f_PPII_*, *Q_net_*, and *N*. These scales, highlighted by the boxed area, predict IDP *R_h_* with good correlation with experimental *R_h_* (R^2^ > 0.7; *x*-axis) and a prediction error that also trends with *Q_net_* (R^2^ > 0.4; *y*-axis). Second, the experimental PPII propensities determined calorimetrically from host–guest analysis of the binding energetics of the Sos peptide (i.e., the peptide-based scale measured by Hilser and coworkers) outperform almost all random scales in their ability to describe sequence effects on mean hydrodynamic size when using only conformational bias and net charge considerations. This is particularly evident when comparing error magnitudes (Figure 6B).

**Figure 7:**
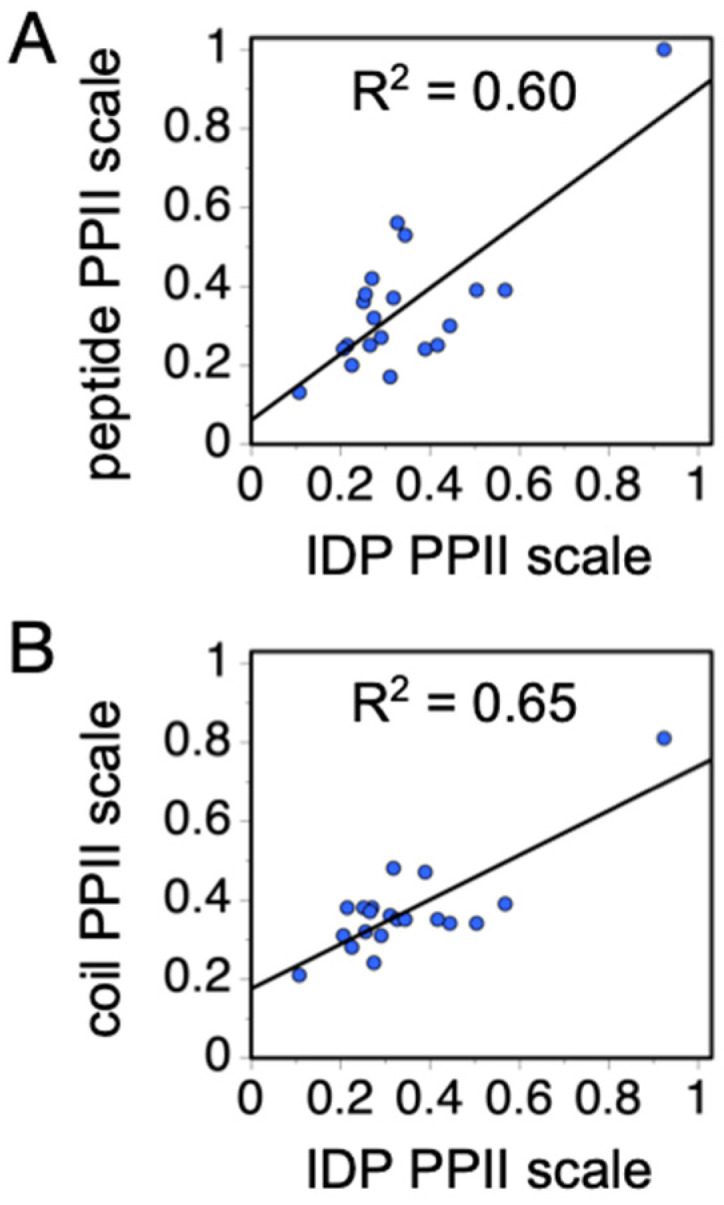
Comparison of experimental PPII propensities. (**A**) Correlation of the peptide-measured PPII scale from Hilser and coworkers (column 4, Table 1) with the IDP-measured PPII propensities (blue bars, Figure 6C). (**B**) Correlation of the coil library (Table 3) and IDP scales. In both plots, each blue circle represents an amino acid type.

To determine if Equation (2) is sufficiently sensitive to discern the differences in PPII bias of the amino acids, the average scale value for each amino acid type was computed from the “best” performing random scales. The “best” scales were defined as those in the boxed area of Figure 6A with the smallest error (i.e., less than the distribution mode; see Figure 6B). The computed averages, unfortunately, report a somewhat trivial specificity except for distinguishing proline and nonproline types (red bars, Figure 6C), most likely owing to the low representation of some amino acid types in the IDP dataset, specifically the nonpolar amino acids [92]. When substitution effects on mean *R_h_* were measured experimentally in p53(1-93) to determine rank order in PPII propensities among the nonpolar amino acid types [37], and then used to restrict the “best” random scales to those that also maintain this rank order, the average scale value by amino acid type (blue bars, Figure 6C) exhibited strong correlations with the other experimental PPII scales (Figure 7). These amino acid-specific average scale values (blue bars, Figure 6C), which were obtained solely from analyzing sequence effects on IDP *R_h_*, represent an independent measure of the intrinsic PPII bias in the ID states of biological proteins.

Because ID has sequence characteristics that show fundamental disparities when compared to nonID sequences, using IDPs as a DSE model for folded protein is not fully supported. For example, unlike the heterogeneous composition of amino acids and the weak repetition found in the sequences of folded proteins [93,94], IDPs and IDRs have a lower sequence complexity [95] with strong preferences for hydrophilic and charged amino acid side chains over aromatic and hydrophobic side chains [92,96]. These disparate properties of the primary sequence suggest potentially disparate structural behavior. To investigate this issue, protein sequence reversal was used to gain experimental access to the disordered ensemble of a protein with a composition of L-amino acids and pattern of side chains identical to those of a conventional folded protein [42]. Using staphylococcal nuclease for these studies, the unaltered wild type adopts a stable native structure consisting of three α-helices and a five-stranded, barrel-shaped β-sheet [97]. The protein variant with reversed sequence directionality, Retro-nuclease, was found to be an elongated monomer, and exhibits the structural characteristics of intrinsic disorder [42]. At 25 °C, the mean *R_h_* of Retro-nuclease was found to be 34.0 ± 0.5 Å by DLS techniques. Sedimentation analysis by analytical ultracentrifugation (AUC) and SEC methods gave similar results under similar conditions (33.0 Å at 20 °C by AUC, and 33.7 Å at ~23 °C by SEC). Equation (2), for comparison, predicts 33.1 Å using the Retro-nuclease primary sequence, which is close to the observed experimental values.

**Figure 8:**
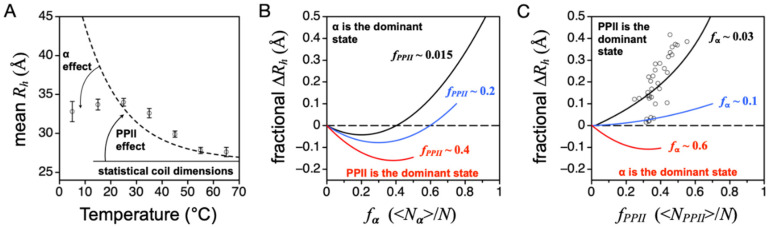
Temperature, α, and PPII effects on DSE hydrodynamic size. (**A**) Open circles show Retro-nuclease mean *R_h_* measured using DLS methods from 5 to 65 °C. The dashed line was calculated with Equation (2) and modeling temperature effects on the intrinsic PPII propensities by Equation (3) and with ∆*H_PPII_* = 13 kcal mol^−1^. Temperature-dependent changes to the amino acid PPII propensities, from Equation (3), cause the Equation (2)-predicted *R_h_* to change accordingly. (**B**,**C**) Simulated effects on population-weighted size from α and PPII bias. Fractional change in mean *R_h_* (i.e., <*L*>/2) was used to normalize simulation results for chain length. In panel C, open circles represent experimental *R_h_* measured for IDPs and normalized relative to the simulated size of an unbiased ensemble [37], as explained in the main text. *f_PPII_* for each IDP was calculated from sequence using the IDP experimental PPII scale (blue bars, Figure 6C).

The hydrodynamic size of Retro-nuclease is highly sensitive to temperature changes (Figure 8A), which is consistent with observations from other IDPs [39,40,41]. The enthalpy and entropy of the PPII to nonPPII transition have been measured in short alanine peptides by monitoring heat effects on structure over a broad temperature range [46]. The results from CD spectroscopy, which monitored the change in the CD signal at 215 nm, gave Δ*H_PPII_* and Δ*S_PPII_* of ~10 kcal mol^−1^ and 32.7 cal mol^−1^ K^−1^, respectively, while NMR measurements, using heat effects on ^3^*J_HNα_*, gave ~13 kcal mol^−1^ and 40.9 cal mol^−1^ K^−1^.

Because the PPII bias is noncooperative [46] and locally determined [72], the effect from temperature changes can be modeled at the level of individual residue positions using the integrated van’t Hoff equation,

(3) $$ln\left(K_{PPII}\left(T\right)\right)=\left(\frac{\Delta H_{PPII}}{R}\right)\left(\frac{1}{\left(298\;K\right)}-\frac{1}{T}\right)+ln\left(K_{PPII}\left(298\;K\right)\right)$$

where *K_PPII_* is the equilibrium between PPII and nonPPII states, *T* is temperature, and *R* is the gas constant. ∆*H_PPII_* is assumed to be constant. If PPII is the lone dominant conformation, then *K_PPII_* for each amino acid type can be estimated from experimental PPII propensities at 25 °C as *K_PPII,i_* = (1 − *PPII_i_*)/*PPII_i_*. The importance of Equation (3) is that it provides another check on the ability of the DSE to be described from the results of peptide studies. Moreover, these two values, Δ*H_PPII_* and *PPII_i_*, give access to the entropy from the relationship (∂*G*/∂*T*)_P_ = −*S*. Using IDP-measured intrinsic PPII propensities (blue bars, Figure 6C), we found that ∆*H_PPII_*~13 kcal mol^−1^ captures the decrease in Retro-nuclease mean *R_h_* from 25 to 65 °C (Figure 8A). For alanine, using its IDP-measured PPII propensity at 25 °C (0.32) and ∆*H_PPII_* = 13 kcal mol^−1^ yields Δ*S_PPII_* = 45.1 cal mol^−1^ K^−1^.

Although the predicted and experimental mean *R_h_* agree at 25 and 65 °C, experimental and Equation (2)-predicted values at 5, 15, 35, and 45 °C show obvious differences (Figure 8A). At 35 and 45 °C, the experimental mean *R_h_* values were larger than predicted, whereas at 5 and 15 °C, they were smaller. The analysis of heat effects on *R_h_* using Equation (3) assumed PPII to be the lone dominant DSE conformation, which is not necessarily correct. Indeed, the Retro-nuclease CD spectrum reported a cold-induced local minimum at 222 nm for *T* < 25 °C [42], revealing temperature-dependent population of the α backbone conformation. By including the effects of an α bias in simulations of DSE hydrodynamic size, both the over- and underpredictions of mean *R_h_* at 5, 15, 35, and 45 °C can be explained. 

Briefly, preferential sampling of main chain dihedral angles for Φ and Ψ associated with α-helix can cause changes in the structural dimensions of the DSE [38]. Monitored from the population-weighted mean size, *R_h_* ~ <*L*>/2, computer-generated ensembles that sample (Φ, Ψ) in the α region (−64° ± 10°, −41° ± 10°) show compaction under modest preferences, and elongated sizes at higher α sampling rates (Figure 8B). Specifically, when (Φ, Ψ) sampling for α is weakly preferred, the probability of contiguous stretches of residues in the α state is low, and turn structures are more likely than helical segments that form when the α bias is higher. Because the effect of the α bias on the mean *R_h_* of the DSE can be accentuated by the PPII bias, whereby ensembles with high PPII propensities show increased sensitivity to changes in the α bias, the consequences of both the α and PPII biases for mean *R_h_* must be considered. For example, the average chain propensity for PPII in our IDP database is ~0.4 when estimated from sequence. Thus, the IDP trend of mean *R_h_* with α bias should follow the red line in Figure 8B, and not the black line. Likewise, the effect of PPII bias on mean *R_h_* is codependent on the α bias (Figure 8C). When PPII is the dominant conformation, the structural dimensions of the denatured state follow the relationship given by Equation (1) (black line in Figure 8C). If, instead, PPII is not the dominant conformation, and moderate α preferences are present, then the *R_h_* dependence on PPII bias changes. More precisely, the result of increasing the chain preference for α is to suppress the effect of PPII on mean *R_h_* (blue line in Figure 8C). When the α bias is stronger than the PPII bias (i.e., α is the dominant conformation), then the effect of the PPII bias is compaction (red line in Figure 8C).

The comparison of experimental IDP *R_h_* to the curves in Figure 8C (open circles in the figure) confirms that PPII is the dominant backbone conformation in IDP ensembles [37]. Here, fractional ∆*R_h_* was calculated as (experimental *R_h_*—simulated *R_h_*)/simulated *R_h_*, where simulated *R_h_* refers to the size of an unbiased ensemble that has been corrected for net charge effects. In the figure, a majority of the IDPs are found to have experimental mean *R_h_* values slightly larger than expected based upon the sequence-calculated value of *f_PPII_*. This suggests that the amino acid preferences for PPII may be underestimated by the IDP-based scale, and the values for *f_PPII_* in this figure should be shifted to the right. The possibility of a larger intrinsic PPII bias cannot be eliminated because PPII effects on mean *R_h_* are suppressed by the presence of an α bias. The magnitude and sequence dependence to the α bias in the protein DSE is currently unknown, although it has been estimated in short alanine-rich peptides [22].

The idea that PPII propensities are underestimated possibly explains some of the Retro-nuclease data shown in Figure 8A. An underestimated PPII bias gives an underestimated predicted mean *R_h_* at 35 and 45 °C. At 5 and 15 °C, the disagreement between theory and experiment is likely caused by the α bias detected in the Retro-nuclease CD spectrum [37,38]. To obtain the sequence dependence of both the α and PPII biases in the DSE and test these assumptions, the analysis of sequence effects on IDP mean *R_h_* reviewed above could simply be repeated at both colder and warmer temperatures. Higher temperatures reduce α effects on mean *R_h_* and isolate the effects of the PPII bias. Colder temperatures give access to the α bias. Just as the sequence dependence of mean *R_h_* at *T* ≥ 25 °C yields the amino acid-specific biases for PPII from the comparison of experimental *R_h_* to simulated coil values that omit PPII effects, the sequence dependence of mean *R_h_* at T < 25 °C can yield the amino acid bias for the α conformation via comparison to the theoretical treatment that omits the α effects.

## 5. Temperature Dependence of Intrinsic α-Helix and PPII Propensities

**Figure 9:**
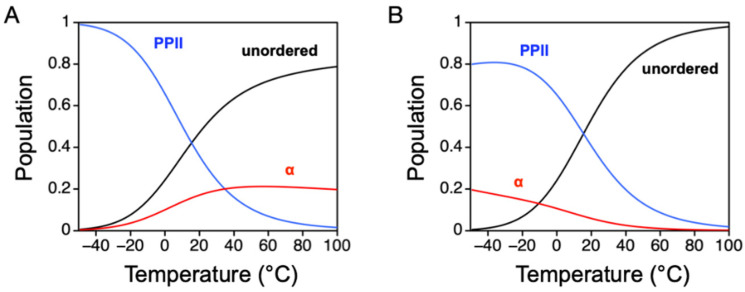
Temperature effects on PPII, α-helix, and unordered populations in an unfolded polypeptide. (**A**) Δ*H_PPII_* = 10 kcal mol^−1^ and ∆*H_α_* = 1 kcal mol^−1^. (**B**) Δ*H_PPII_* = 10 kcal mol^−1^ and ∆*H_α_* = 11 kcal mol^−1^. To model a generic polypeptide, the PPII and α-helix propensities used the average value from the propensities measured by Hilser and coworkers (column 4, Table 1) and Baldwin and coworkers (column 3, Table 2). Specifically, the PPII propensity was 0.35 at 25 °C, while the α-helix propensity was 0.29 at 0 °C. To calculate populations, the partition function was determined from *Q* = 1 + e^−∆*GPPII/RT*^ + e^−∆*Gα/RT*^, with the unordered state as the reference. ∆*G_PPII_* and ∆*G_α_* were calculated from the propensities by *−RTln*(*PPII*/1 − *PPII*) and −*RTln*(*α*/1 − *α*), and the temperature dependence of the propensities was calculated with Equation (3). The unordered, α-helix, and PPII populations thus were 1/*Q*, e^−∆*Gα/RT*^/*Q*, and e^−∆*GPPII/RT*^/*Q*.

If we assume Tiffany and Krimm are correct, and the DSE is composed of three main structural states (PPII, α-helix, and unordered), then the PPII and α-helix propensities given in Table 1 and Table 2 can be used to model how PPII, α-helix, and unordered populations change with temperature for a generic polypeptide. This is shown in Figure 9A, where populations at different temperatures were modeled by using the integrated van’t Hoff equation (Equation (3)), a transition enthalpy for PPII to nonPPII (Δ*H_PPII_*), and a transition enthalpy for α to non-α (∆*H_α_*). As discussed above, peptide [46] and IDP studies [37,42] both indicate Δ*H_PPII_* is ~10 kcal mol^−1^. Calorimetric studies using alanine-rich peptides that adopt α-helix by Bolen and coworkers indicate ∆*H_α_* is ~1 kcal mol^−1^ [98]. In this model, because Δ*H_PPII_* >> ∆*H_α_*, PPII populations are highly sensitive to temperature changes, while α-helix populations show reduced temperature sensitivity. Moreover, also because Δ*H_PPII_* >> ∆*H_α_*, PPII populations dominate at very cold temperatures. Unfortunately, the model predicts α-helix populations that decrease with decreasing temperatures, in stark contrast to the known stabilities of peptide and protein structures.

If, instead, ∆*H_α_* is given a value comparable to Δ*H_PPII_*, the model yields temperature-dependent populations that reasonably agree with experimental results (Figure 9B). Specifically, both PPII and α-helix populations decrease to low levels at high temperatures. Moreover, under cold conditions, PPII dominates, but α-helix is also populated at non-negligible levels that gradually increase as heat is removed from the system. This result from the model can be explained by assuming that the calorimetry measured ∆*H_α_* is the net heat associated with forming α-helix at the cost of disrupting PPII (i.e., ∆*H_α_*~∆*H_cal,α_* + Δ*H_PPII_*~1 kcal mol^−1^ + 10 kcal mol^−1^ = 11 kcal mol^−1^). In Figure 9B, the transition enthalpies are modeled as 10 kcal mol^−1^ and 11 kcal mol^−1^ for Δ*H_PPII_* and ∆*H_α_*, respectively. This model is supported by the experimental data obtained for Retro-nuclease (Figure 8). The observed temperature dependence of the Retro-nuclease hydrodynamic size revealed PPII and α-helix intrinsic propensities that changed with temperature in a manner similar to the Figure 9B model.

## 6. Discussion

Structural and energetic characterization of the DSE is required for a molecular-level understanding of both protein stability and fold specificity. Historically, short peptides [11,12,13] and the protein coil library [27,28,29,30] have been used as the principal models from which to investigate the DSE. For these two models, there is good quantitative agreement in the sense that the protein coil library, when compared to peptide results, has been found to reproduce the intrinsic conformational preferences of the amino acids for helix, sheet, and PPII [29], as well as the effects on the conformational preferences from neighboring residues [31]. This agreement between two independent models indicates that the magnitudes and types of intrinsic biases in unstructured polypeptides are reasonably well-known. The role of the temperature in describing DSE structure, however, is less well understood. Heat indeed modulates the populations of unstructured states, which is evidenced by the large temperature-dependent changes in hydrodynamic size exhibited by IDPs [39,40,41]. Moreover, the ability of a protein to fold [2], phase separate [99], or recognize its binding partner [69] is also temperature-dependent.

Recently, we demonstrated that the enthalpy, entropy, and magnitude of DSE conformational bias can be elucidated by analyzing heat effects on the mean *R_h_* of IDPs [37]. The sequence dependence of IDP hydrodynamic size yields an independent measure of the intrinsic bias for PPII, because PPII-rich structures are extended [43]. Additionally, as the PPII bias is driven by a favorable enthalpy [46], the effect of increased temperature is to populate nonPPII states at the expense of PPII. Thus, the enthalpy and entropy of the PPII–nonPPII transition can be determined from the heat-induced changes to the mean *R_h_*. Our analysis of the sequence dependence on IDP hydrodynamic size revealed amino acid-specific preferences for PPII that are in good quantitative agreement with both calorimetry-measured values from short peptides and those inferred by a survey of the protein coil library (Figure 7). Modeling the effects of heat on IDP hydrodynamic size yields an enthalpy and entropy of PPII formation that were quantitatively similar to the peptide-measured values [37,46]. It is important to note that these three DSE models (i.e., peptides, the coil library, and IDPs) universally report that the allowed regions of Ramachandran space are unevenly sampled, and that PPII is the predominant denatured state conformation under normal conditions.

When interpreting the effects of the PPII bias on the mean *R_h_* of unstructured proteins, the population of the α backbone conformation has consequences that must be considered. The α basin of the Ramachandran map of Φ and Ψ dihedral angles is among the most populated regions in the coil library distribution [27,30], and is shared with turn structures [29]. Because of the backbone geometry of the α configuration, whereby sparse sampling at dispersed positions can produce turns, and heavy sampling among contiguous positions yields helices, the effect of a PPII bias on the mean *R_h_* can be either compaction or expansion. This is demonstrated in Figure 8C. The codependence of DSE mean *R_h_* on both the α and PPII biases predicts that intrinsic α preferences, and its corresponding thermodynamic parameters, can be estimated from low-temperature studies that compare experimental *R_h_* to computer-simulated DSE trends (Figure 8A). Specifically, for some unstructured proteins, the intrinsic α bias at low temperatures may be sufficiently strong that its magnitude, sequence dependence, and enthalpy and entropy of formation can be measured from the effect on the mean *R_h_*. It remains to be seen if this strategy can be successful, and if the resultant intrinsic α propensities as measured in IDPs compare favorably to those obtained from short peptides (Table 2) and surveys of the protein coil library [27,28,29,30].

## Figures and Tables

**Table 1:** Experimental intrinsic propensity for the PPII backbone conformation measured in short peptides.

Amino Acid	PPII Propensity ^a^	PPII Propensity ^b^	PPII Propensity ^c^
ALA (A)	0.61	0.818	0.37
CYS (C)	0.55	0.557	0.25
ASP (D)	0.63	0.552	0.30
GLU (E)	0.61	0.684	0.42
PHE (F)	0.58	0.639	0.17
GLY (G)	0.58	-	0.13
HIS (H)	0.55	0.428	0.20
ILE (I)	0.50	0.519	0.39
LYS (K)	0.59	0.581	0.56
LEU (L)	0.58	0.574	0.24
MET (M)	0.55	0.498	0.36
ASN (N)	0.55	0.667	0.27
PRO (P)	0.67	-	1.00
GLN (Q)	0.66	0.654	0.53
ARG (R)	0.61	0.638	0.38
SER (S)	0.58	0.774	0.24
THR (T)	0.53	0.553	0.32
VAL (V)	0.49	0.743	0.39
TRP (W)	-	0.764	0.25
TYR (Y)	-	0.630	0.25
average	0.58	0.626	0.35

^a^ Measured at the X position in Ac-(Pro)_3_-X-(Pro)_3_-Gly-Tyr-NH_2_ by Creamer and coworkers, at 5 °C, and excluding Tyr and Trp [12]. ^b^ Measured at the X position in Ac-(Gly)_2_-X-(Gly)_2_-NH_2_ by Kallenbach and coworkers, at 20 °C, and excluding Gly and Pro [16]. ^c^ Measured at the X position in Ac-Val-(Pro)_2_-X-Val-(Pro)_2_-(Arg)_3_-Tyr-NH_2_ by Hilser and coworkers, at 25 °C [17].

**Table 2:** Experimental intrinsic propensity for the α-helix measured in short peptides.

Amino Acid	∆*G* (kcal mol^−1^) ^a^	α-Helix Propensity ^b^
ALA (A)	−0.258	0.62
CYS (C)	0.570	0.26
ASP (D)	0.635	0.24
GLU (E)	0.433	0.31
PHE (F)	0.672	0.22
GLY (G)	1.62	0.05
HIS (H)	0.525	0.28
ILE (I)	0.445	0.31
LYS (K)	0.108	0.45
LEU (L)	0.022	0.49
MET (M)	0.251	0.39
ASN (N)	0.635	0.24
PRO (P)	4	0.001
GLN (Q)	0.314	0.36
ARG (R)	−0.047	0.52
SER (S)	0.525	0.28
THR (T)	1.07	0.12
VAL (V)	0.797	0.19
TRP (W)	~0.6	0.25
TYR (Y)	~0.4	0.32
average		0.29

^a^ Measured in an alanine-rich host at 0 °C by Baldwin and coworkers [22]. In the original report, bias for the α-helix was given as a free energy (∆*G*) of helix formation. The values for ASP, GLU, LYS, and ARG represent the charged species; His value is for the neutral species. ^b^ α-helix propensities were estimated from the free energies as *K_α_*/(1 + *K_α_*), where *K_α_* = e^−∆*G*/*RT*^, ∆*G* is from column 2, *R* is the gas constant, and *T* is temperature.

**Table 3:** Amino acid specific propensity for the PPII backbone conformation in the protein coil library.

Amino Acid	PPII Propensity ^a^
ALA (A)	0.48
CYS (C)	0.38
ASP (D)	0.34
GLU (E)	0.38
PHE (F)	0.36
GLY (G)	0.21
HIS (H)	0.28
ILE (I)	0.39
LYS (K)	0.35
LEU (L)	0.47
MET (M)	0.38
ASN (N)	0.31
PRO (P)	0.81
GLN (Q)	0.35
ARG (R)	0.32
SER (S)	0.31
THR (T)	0.24
VAL (V)	0.34
TRP (W)	0.35
TYR (Y)	0.37
average	0.37

^a^ Calculated by Freed and coworkers using a restricted coil library that removed α-helices, β-sheets, turns, and residues flanking secondary structures from a set of protein structures [29].

## Data Availability

Data presented in this study are openly available and cited in the references.
